# Strains, Mechanism, and Perspective:* Salmonella*-Based Cancer Therapy

**DOI:** 10.1155/2016/5678702

**Published:** 2016-04-14

**Authors:** Cheng-Zhi Wang, Robert A. Kazmierczak, Abraham Eisenstark

**Affiliations:** ^1^National Laboratory of Biomacromolecules, Institute of Biophysics, Chinese Academy of Sciences, Beijing 100101, China; ^2^Cancer Research Center, Columbia, MO 65201, USA; ^3^University of Missouri-Columbia, Columbia, MO 65201, USA

## Abstract

Recently, investigation of bacterial-based tumor therapy has regained focus due to progress in molecular, cellular, and microbial biology. Many bacteria such as* Salmonella*,* Listeria*,* Escherichia*, and* Clostridium* have proved to have tumor targeting and in some cases even tumor-destroying phenotypes. Furthermore, bacterial clinical treatments for cancer have been improved by combination with other therapeutic methods such as chemotherapeutic drugs and radioactive agents. Synthetic biology techniques have also driven the development of new bacterial-based cancer therapies. However, basic questions about the mechanisms of bacterial-mediated tumor targeting and destruction are still being elucidated. In this review, we focus on three tumor-therapeutic* Salmonella* models, the most intensively studied bacterial genus in this field. One of these* Salmonella* models is our* Salmonella enterica* serovar Typhimurium LT2 derived strain CRC2631, engineered to minimize toxicity but maximize tumor-targeting and destruction effects. The other two are VNP20009 and A1-R. We compare the means by which these therapeutic candidate strain models were selected for study, their tumor targeting and tumor destruction phenotypes* in vitro* and* in vivo*, and what is currently known about the mechanisms by which they target and destroy tumors.

## 1. Introduction to Bacterial Tumor Therapy

Two centuries ago, there were reports that cancer patients went into remission after recovering from bacterial infections [1]. From the late 19th to early 20th century, William Coley, an American physician, did a series of experiments to treat his cancer patients with both live and heat-killed bacteria such as* Streptococcus pyogenes* and* Serratia marcescens*. The combination of Coley's heat-killed bacteria was referred to as “Coley's toxin” and remained in clinical use for sarcomas patients until 1963 [1]. In 1976, BCG (Bacillus Calmette-Guérin) bacteria were reported to successfully treat superficial bladder cancer by stimulation of the immune system and inflammatory response [2]. This therapy is still in clinical use [3].

Recently, many types of bacteria have been found or developed to either target or destroy solid tumors in animal models or human clinical trials. Yazawa et al. [4] reported in 2001 that* Bifidobacterium longum*, a nonpathogenic and anaerobic strain, selectively localized to and grew in 7,12-dimethylbenzanthracene-induced rat mammary tumors after systemic injection. Dang et al. [5] assessed the efficacy of several anaerobes in their tumor therapeutics. Among the 26 species belonging to the* Clostridium, Lactobacillus*, and* Bifidobacterium* genera tested, two strains,* Clostridium novyi* and* Clostridium sordellii*, were found to exhibit extensive tumor localization, especially in poorly vascularized areas. The* C. novyi* strain was attenuated by removing the *α*-toxin to generate a nontoxic strain named* C. novyi*-NT. Therapeutic administration of this strain in combination with conventional chemotherapy or radiotherapy was highly effective in animal tumor models. For instance, the combination of* C. novyi*-NT spores and liposomal doxorubicin resulted in complete regression of tumors in all treated mice [6]. However, this strain grows only in the deep hypoxic part of the tumors, leaving the oxygenated rim area uncolonized, which results in tumor recurrence.

Facultative anaerobic bacteria such as* Salmonella, Escherichia, Shigella, Vibrio*, and* Listeria* can overcome this problem to some extent. They have the capability to target and colonize small (nonhypoxic) and quiescent (hypoxic) tumors as well as metastatic tumor regions accessible by the circulatory system. Yu et al. [7] tested a broad range of bacteria on different tumor models including syngeneic, xenogeneic, and spontaneous tumors in mice and found that most of these species were able to colonize and replicate preferentially in tumors but with varying levels of efficiency. The same group tested pathogenic* Salmonella enterica* serovar Typhimurium (hereafter* S. typhimurium*) strains ATCC14028 and SL1344, attenuated* Shigella flexneri* strain 2a SC602,* Escherichia coli* (*E. coli)* strain 4608-58, uropathogenic* E. coli* strain CFT073, the nonpathogenic* E. coli* strain Top 10, and the probiotic* E. coli* strain Nissle 1917 in syngeneic 4T1 tumor-bearing BALB/c mice. All these strains exhibited high tumor colonization ability. Investigators recovered more than 1 × 10^8^ colony-forming units (CFU) per gram tumor tissue [8]. Among these tested strains,* E. coli* showed the most robust tumor-specific colonization with negligible colonization of spleen and liver. The* E. coli* Nissle 1917 strain showed similar colonization and amplification in tumors of immunocompetent and immunocompromised animals [8]. A recent phase I clinical trial was performed using a live-attenuated* Listeria monocytogenes* (*L. monocytogenes*) vaccine in advanced cervix carcinoma patients who failed to respond to chemotherapy, radiotherapy, and surgery [9]. The results are very promising showing that median survival time in these patients is doubled by the bacterial administration.

## 2. *Salmonella*: Bacterial Tumor-Targeting Model and Therapy

*Salmonella* species are by far the most extensively studied bacteria in the field of tumor targeting. They are Gram-negative facultative anaerobic bacteria that can grow and replicate inside host cells. Some* Salmonella* strains have the intrinsic property to preferentially colonize solid tumors [10–13]. Several* Salmonella* strains were tested for their antitumor effects several decades ago. Today, there are multiple* Salmonella* strains being developed for targeted chemotherapy delivery, notably VNP20009, A1-R, and CRC2631.

### 2.1. VNP20009

The most intensively studied tumor-targeting* Salmonella* strain is VNP20009, which was derived from* S. typhimurium* ATCC14028. VNP20009 is a genetically attenuated strain developed at Yale University that possesses an excellent safety profile and is derived from* S. typhimurium* ATCC14028. This safety profile includes genetically attenuated virulence (a deletion in the* purI* gene), reduction of septic shock potential (a deletion in the* msbB* gene), and antibiotic susceptibility [12]. This strain keeps its genetic and phenotypic stability after multiple generations both* in vitro* and* in vivo* [12]. In tumor-bearing mice, VNP20009 accumulates preferentially in transplanted murine tumors and in a variety of human tumor xenografts over normal organs at a ratio of >1000 : 1.* In vivo* experiments of this strain in nonhuman primates demonstrated that VNP20009 is cleared from the blood in 24 hours and completely cleared from all organs in monkey models by day 41 [12]. VNP20009 is the only* Salmonella* strain to be evaluated in a phase I clinical trial for treatment of nonresponsive metastatic melanoma or renal cell carcinoma in humans [14]. Single doses of VNP20009 ranging from 1 × 10^6^ CFU/m^2^ to 1 × 10^9^ CFU/m^2^ were administrated into cohorts of three to six patients. Five of these subjects had stable responses to the initial bacterial injection and received a second cycle of VNP20009 at the same dose level. Significant quantities of IL-1*β* and TNF-*α* were initially detected in the serum after introduction of VNP20009 into subjects. Other cytokines such as IL-6 and IL-12 were also induced by VNP20009. Focal tumor colonization and necrosis were observed in two patients receiving 1 × 10^9^ CFU/m^2^ (dose-limiting toxicity) and in one patient receiving 3 × 10^8^ CFU/m^2^ (the maximum-tolerated dose). However, although VNP20009 colonization resulted in localized necrosis of tumors, none of the patients treated with VNP20009 showed any evidence of tumor regression [14]. These results suggested that this strain needed to either reduce dose-related toxicity so more VNP20009 could be introduced and/or improve localization of VNP20009 to tumor sites so that dosage levels could colonize tumors more effectively. Some modifications have been introduced to this strain to enhance safety and/or antitumor phenotypes. For example, Cheng et al. [15] deleted the* phoP*/*phoQ* system of VNP20009. The modified strain exhibited lower titers in tumor-free livers and spleens compared with the original VNP20009 strain. Also, its tumor-targeting ability was significantly enhanced [15]. Recently, the complete genome of VNP20009 has been sequenced [16]. Besides the known* purI* and* msbB* gene deletions, other mutations have also been found, including* purM* deletion, a 108 kb Suwwan deletion, and 50 nonsynonymous SNPs [16]. Biological significance of these mutations awaits further investigation.

### 2.2. A1/A1-R

Another* S. typhimurium* ATCC14028 tumor-targeting strain that selectively grew in tumor xenografts (A1) was developed at University of California (San Diego) by treating* S. typhimurium* with nitrosoguanidine (NTG) to induce mutations and the resulting auxotrophic pool selected by ability to grow in successive tumor xenografts [13].* S. typhimurium* strain A1 was recovered from this selection and identified as a leucine and arginine auxotroph. Presumably, the auxotrophic strains can receive sufficient amounts of these amino acids from the tumor environment but do not persist in the normal tissue environment.* In vitro* experiments showed that A1 could invade and replicate intracellularly in PC-3 human prostate cancer cell line and as few as 10–50 CFU bacteria could induce cytopathic effects in the PC-3 cells. Safety tests showed that all mice survived after intravenous injection of 10^7^ CFU A1 while all mice died in 3 days using the ATCC14028 wild-type bacteria challenge group. The A1 auxotroph can selectively target the PC-3 tumor* in vivo* with tumor : liver bacterial ratios as high as 2,000–10,000 : 1 by day 4 with* Salmonella* completely clearing from normal tissue after approximately 2 weeks. Colonization of the PC-3 tumor by strain A1 suppressed its growth, although it failed to destroy the tumor completely. Direct intratumoral injection of* S. typhimurium* A1 resulted in the subcutaneous PC-3 tumor completely disappearing within 15–26 days after the start of treatment [13]. To further improve tumor-targeting capability and tumor toxicity,* S. typhimurium* A1 was passaged by injection in nude mice transplanted with the HT-29 human colon tumor and reisolated as A1-R [17]. After reselection, the number of A1-R bacteria attached to HT-29 human colon cancer cells was approximately sixfold higher than parental A1 [17]. Enhanced tumor virulence showed that all PC-3 human prostate cancer cells were infected and lysed within 200 minutes by A1-R* in vitro* and A1-R had 100 times more CFU in PC-3 tumor tissue than A1* in vivo* [18]. Forty percent of all orthotopic metastatic human prostate tumor-bearing mice were completely cured by weekly A1-R bacterial treatment [18]. This strain has been tested in many other primary cancers as well, including prostate, breast, pancreatic, and spinal-cord tumor models, as well as some metastatic tumors, and the results are promising [19–22]. Very recently, A1-R was shown to be capable of decoying quiescent cancer cells to S/G2/M phase and sensitize them to cytotoxic chemotherapy, further expanding its therapeutic potential [23].

### 2.3. CRC2631

**Figure 1:**
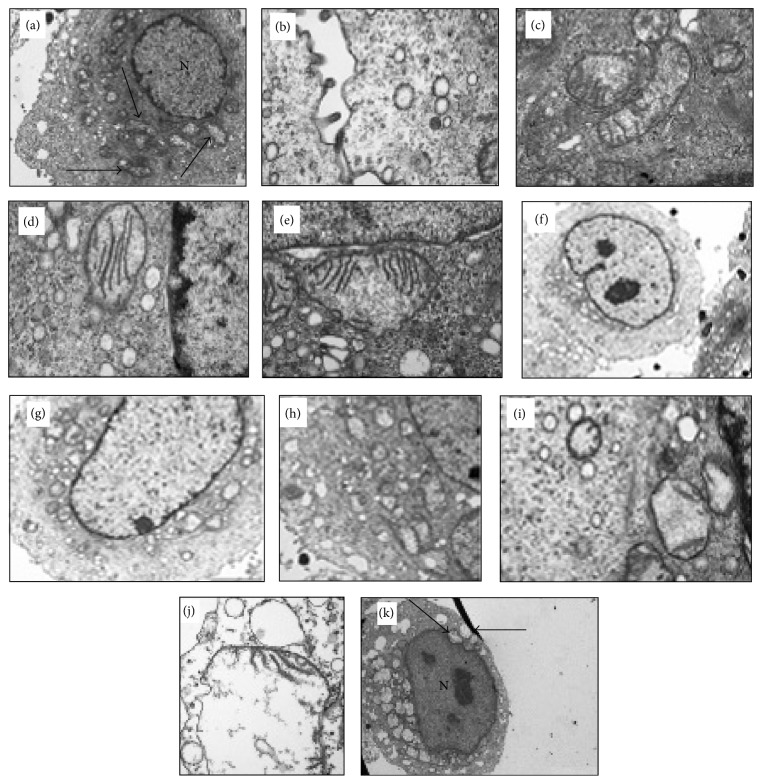
TEM of* S. typhimurium* at 1 hr (a–e), 4 hrs (f–i), and 8 hrs (j and k) displaying various degrees of mitochondria destruction as a result of* Salmonella* infestation. In (a), mitochondria are shown (arrows) in which most of the cristae are destroyed. (c) through (e) show mitochondria with degraded cristae. (k) shows mitochondria within the PC-3M cell in which mitochondria (arrows) appear to be empty while the nucleus (labeled “N”) does not appear to be affected [10].

CRC2631 is a tumor-targeting* Salmonella* strain model developed at the Cancer Research Center (Columbia, MO) and is a candidate therapeutic derived from the* S. typhimurium* LT2 wild type [24]. This strain was developed using archived* Salmonella* strains from the original Demerec collection of LT2 auxotrophs [25]. These strains have been stored in agar stabs for more than four decades at room temperature and have generated dramatic genetic diversity including deletions, duplications, frameshifts, inversions, and transpositions [25, 26]. Coupled with their original auxotrophies, these mutations made the collection an ideal pool for selection of nontoxic therapeutic* Salmonella*. The Demerec collection strains were screened by coincubating them with MCF-7 human breast cancer cells and comparing the targeting phenotypes with the ATCC14028-derived* Salmonella* therapeutic strain VNP20009. We found one strain, CRC1674, which targeted and destroyed breast cancer cells more effectively than results obtained with VNP20009. Genetic investigation indicates that CRC1674 contains numerous mutations including* his*-2550 (plus suppressor mutation, DIIR49B), altered* rpoS* start signal (UUG), G to T mutation in position 168 in* rpoS* sequence, and decreased HPI and HPII [24]. CRC1674 was further engineered to disrupt* aroA*,* thyA*, and* rfaH* to generate an LPS-deficient strain auxotrophic for biosynthesis of aromatic amino acids and thymine. The attenuated strain, CRC2631, did not change its tumor-targeting and destroying phenotype but decreased its toxicity dramatically. CRC2631 preferentially targets PC-3M human prostate cancer cells compared to RWPE-1 normal prostate cell line at ratios from >200 : 1 to >1000 : 1. Coincubation of CRC2631 and human prostate cancer cell line PC-3M results in colonization of PC-3M and destruction of their mitochondria within one hour (Figure 1) [10]. Up to 1.2 × 10^8^ CFU of CRC2631 can be tolerated in TRAMP mice (a mouse model which spontaneously develops autochthonous prostate tumors), showing its safety in mammalian hosts. When the TRAMP prostate cancer mouse model was intraperitoneally injected with 1 × 10^7^ CFU, the ratio of* Salmonella* counts were up to 100-fold greater in the TRAMP mouse prostate tumor masses versus the usual* Salmonella* reservoirs of the liver and spleen after 72 hours (Kazmierczak et al., unpublished data). Strain CRC2631 recovered from TRAMP mouse prostate tumors showed significant loss of wild-type motility and flagella, indicating phenotypic evolution of this therapeutic strain within the tumor environment [27]. Further optimization of CRC2631 is under way.

## 3. Mechanisms of* Salmonella*-Tumor Interaction

The interaction between* Salmonella* and host epithelial cells in the GI tract, as well as* Salmonella*-macrophage interactions, has been researched extensively in the past two decades. However, little is known about the unique interaction features between* Salmonella* and tumor cells. Understanding the mechanism of how* Salmonella* preferentially targets cancer cells and colonizes tumors will not only help us to improve therapeutic strains but also deepen our comprehension of tumor biology. Key questions in this field include what characteristics of tumors are required to accept* Salmonella* colonization, as well as why* Salmonella* does not preferentially target, invade, and kill nonmalignant cell lines. Finally, understanding* Salmonella*'s tumor-targeting mechanism(s) will dramatically accelerate therapeutic strain development by allowing researchers to design and engineer tumor-seeking mechanisms directly.

### 3.1. Targeting Cancer Cell Components

One of the first questions asked is why these* Salmonella* specifically migrate to the tumor region. It is reasonable to hypothesize that there are some unique or highly expressed molecules generated by tumors that attract* Salmonella* toward them via chemotaxis. Kasinskas and Forbes [28] have found that* Salmonella* is attracted to an* in vitro* tumor cylindroid model, accumulating in the central regions of the quiescent tumor cell mass. In this model, the presence of necrotic and quiescent cells enabled* Salmonella* strains to replicate in tumor tissue. Further research demonstrated many mechanisms essential for this phenotype including the aspartate receptor on the* Salmonella* surface, which initiated chemotaxis toward tumor cylindroids* in vitro*, the serine receptor, which initiated penetration of the cylindroid, and the ribose/galactose receptor, which attracted* S. typhimurium* toward necrotic tissue. Strains lacking proper flagella constructs (*fla*), signal transduction proteins (*cheA, cheY*), or active motor function (*mot*) lose the ability to chemotax toward tumor cylindroids, suggesting that directed chemotaxis is necessary to promote accumulation in tumors. However, these results have not been proven* in vivo* [11].

Our laboratory investigated whether human glycoproteins, defined as glycan binding proteins with two to six linear monosaccharides (with or without side chains), were serving as chemoattractants to a tumor-targeting* Salmonella* strain. The human glycome is not yet fully defined but includes cellular receptors, signaling molecules, and enzymes that may be uniquely or preferentially expressed by cancer cells. It is thought that there may be approximately 3000 glycan determinants with an additional ~4000 theoretical pentasaccharide sequences in glycosaminoglycans [29]. In order to determine whether* Salmonella* specifically recognized a unique or specific class of glycan determinant(s) expressed by human cells, we coincubated multiple* Salmonella* strains (LT2, VNP20009, CRC1674, and CRC2631) with 285 glycans purified from human tissues and spotted onto glass arrays. We observed that all the* Salmonella* strains bound approximately 5% (fourteen) of the human glycans at levels from 10 to >400 times more efficiently than background levels. We performed literature searches of the glycans preferentially bound by* Salmonella*. Publications showed preferential expression of* Salmonella*-bound glycoproteins identified on the array above by human neoplastic tissues [30] and upregulated expression of GlcNAc*β*1-4GlcNAc*β*-N/Gly terminal disaccharides on various carcinoma cells [31], demonstrating that these glycoproteins are highly expressed in certain human cancer cell lines and represent a mechanism by which* Salmonella* can successfully target and bind to tumor cells. Realizing that we have only tested a small, nonspecific pool of human glycoproteins, current work in our lab includes isolation and profiling of cancer glycoproteins to identify specific* Salmonella*-glycoprotein binding associations.

### 3.2. Bacterial Colonization Using Tumor-Specific Architecture

A passive mechanism that enhances but is not required for* Salmonella* tumor colonization has been observed* in vivo* by the Weiss group [32]. In this model, a rapid increase of TNF-alpha, as well as other proinflammatory blood cytokines, was observed soon after intravenous administration of* S. typhimurium* into tumor-transplanted mice. This was followed by tremendous influx of blood into the tumors containing the endothelial disruptions (larger pores, gaps) typical of tumor blood vessel architecture. Blood-borne bacteria might be flushed in with this influx. Depletion of TNF-alpha results in blood influx retardation and delayed bacterial tumor colonization. These results suggest that blood-borne* Salmonella* are passively flushed into the tumor regions by the blood flow. This may explain the poor outcome of phase I clinical trials of VNP20009, which failed to induce sufficiently high TNF-alpha concentrations spikes in the human body, thus hampering* Salmonella* entry into tumor tissues.

### 3.3. *Salmonella*/Immune System Interactions

The host immune system also plays an important role in the regulation of* Salmonella*-tumor interaction* in vivo*. Bacteria can promote antitumor immunity as potent natural adjuvants by activating innate immune cells followed by secretion of cytokines that can recruit and activate other immune cells at the tumor site [33]. On the other hand,* Salmonella* may grow or persist inside necrotic and hypoxic areas due to destruction by host neutrophils [34]. Strikingly, Crull et al. [35] found that, in solid CT26 tumors,* S. typhimurium* SL7207 resides extracellularly and produces biofilms in response to host neutrophils, protecting them from uptake by phagocytic as well as tumor cells. Mutant strains lacking key biofilm regulators such as* adrA* and* csgD* can be found intracellularly, however, with delayed tumor colonization [35]. This discovery suggests* Salmonella* reactions on human immune system must be considered in further therapeutic approaches.

*Salmonella enterica* serovar Choleraesuis (*S. choleraesuis*) was administrated into T-cell-deficient and B-cell-deficient tumor-bearing mice models to test the roles of immune cells in* Salmonella*-tumor interaction [36, 37]. Normally, notable increases of CD8+ and CD4+ T cells infiltrated into tumors in immunocompetent mice treated with* S. choleraesuis*. Tumor inhibition and infiltration of macrophages and neutrophils were lower in T-cell-deficient mice, proving that T cells are required for the wild-type tumor inhibition phenotype. It was also found that* S. choleraesuis* significantly upregulates interferon-*γ* in wild-type and CD8+ T-cell-deficient mice, but not in CD4+ T-cell-deficient mice. In the B-cell-deficient mice model, the bacterial loads of healthy organs were higher than those in wild-type mice, indicating that B cells function as a border that can inhibit* Salmonella* dissemination into normal organs.

### 3.4. Selection and Engineering of Improved Tumor-Targeting* Salmonella*

With the fast development of molecular biology, high throughput methods have been introduced into research on* Salmonella*-tumor interaction. Arrach et al. [38] cloned a random library of* S. typhimurium* ATCC14028 genomic DNA upstream of a promoterless GFP gene. A* Salmonella* pool containing this library was administrated into PC-3 prostate tumor mice models as well as normal nude mice control. Tumor-activated but not spleen-activated promoters were sorted by FACS and analyzed by oligonucleotide tiling array. Eighty-six intergenic regions were enriched specifically in tumor. Another* Salmonella* transposon insertion mutant pool was tested, selecting for mutants that showed reduced fitness in normal tissues but retain unchanged fitness in tumors [39]. Recently, Yu et al. [40] engineered a* S. typhimurium* strain SL7207 to an obligate anaerobic strain by placing an essential gene under a hypoxia-conditioned promoter. This strain exhibited enhanced tumor-targeting ability and significant decreased toxicity on both immunocompromised and immunocompetent mouse xenograft tumor model [40, 41]. These examples have potential practical importance for engineering better therapeutic* Salmonella* strains.

## 4. Engineered Tumor-Targeting* Salmonella* as Therapeutic Vectors

Although some* Salmonella* strains possess native bacterial cytotoxicity against tumors, it is obvious that addition of toxins may enhance their antitumor effects.* Salmonella* has the advantage of being easy to engineer since it has long been a bacterial genetic model. Currently, the most popular strategies are (1) expressing enzymes to activate anticancer “prodrugs” at tumor sites; (2) expressing anticancer agents directly; (3) expressing tumor-specific antigens and antibodies; (4) transferring eukaryotic expression vectors into tumor cells; and (5) expressing oncogene silencing RNA [42–44]. Some other strategies, such as delivery of tumor-killing nanoparticle therapies, are still being developed.

### 4.1. Expressing Prodrug Converting Enzymes to Release Drugs in Tumor Sites

Prodrugs are biologically inactive compounds that can be metabolized in the body to produce an active drug. To avoid the systematic toxicity from traditional nontargeted cytotoxic chemotherapy, many suicide gene therapies using a variety of delivery vectors have been introduced to exclusively express prodrug converting enzymes inside or close to tumor cells.* Clostridium*,* Bifidobacteria*,* Lactobacillus*, and* Caulobacter* have been investigated to activate prodrugs in the hypoxic region of the necrotic center of tumors [5, 45–47].* Salmonella* has also been used to deliver prodrug-activating enzymes. Pawelek et al. [48] have used* S. typhimurium* to deliver herpes simplex virus thymidine kinase (*HSV* TK) with a beta-lactamase secretion signal to phosphorylate the prodrug ganciclovir (a nucleoside analogue). Better tumor retardation and prolonged survival were observed with* Salmonella*/ganciclovir treatment compared with bacteria alone. Fu et al. [49] have used* S. typhimurium* containing the* E. coli* purine nucleoside phosphorylase (ePNP) gene, which can convert MoPdR into methoxypurine. Combination therapy significantly inhibited tumor growth by approximately 86.6–88.7% and prolonged the survival of tumor-bearing mice. VNP20009 has been engineered to express prodrug-activating enzyme carboxypeptidase G2 (CPG2) to significantly reduce the growth of xenografts compared to using the therapeutic strain alone [50]. Other prodrug converting enzymes such as cytosine deaminase (which can convert 5-fluorocytosine (5-FC) to 5-fluorouracil (5-FU)) have also been introduced to* Salmonella* vectors [51].

### 4.2. Expressing Anticancer Agents

Bacterial toxins were first used to combat cancer as early as over one hundred years ago when William Coley used his “Coley's toxin” [1]. Increasing categories of bacterial toxins have been introduced to tumor-targeting* Salmonella* strains. For instance, colicin E3 (ColE3) and HlyE (also known as ClyA) have both shown increased tumor-killing ability when expressed in* S. typhimurium* [52, 53].* Salmonella* expressing TNF-alpha family cytotoxic agents such as TNF-alpha, FAS ligand (FASL), and TNF-related apoptosis-inducing ligand (TRAIL) can enhance cytotoxic effects against many kinds of tumors [54–56]. Deficiencies of these cytotoxic agents, such as hepatotoxicity and short circulatory half-life, can be overcome by* Salmonella*-mediated targeting to the tumor site and creating highly localized concentrations of anticancer agents [42]. Immunomodulatory molecules such as cytokines and chemokines are able to stimulate the host immune system to clear tumors. These molecules can be delivered by* Salmonella* vector and have been proven to inhibit tumors. For instance, IL-2 has been extensively studied in tumor-targeting* Salmonella* expressing systems [57–61]. Enhanced tumor regression or tumor growth inhibition by the combination of* Salmonella* with IL-2 compared with* Salmonella* alone has been verified* in vivo*. Other cytokines such as IL-18, LIGHT, and CCL21 have also been found to inhibit both primary and metastatic tumors when expressed by* Salmonella* vectors [55, 62, 63].

### 4.3. Expressing Tumor-Specific Antigens and Antibodies

Bacteria can be engineered to express numerous tumor-specific antigens that sensitize the host immune system to prevent tumor formation or inhibit tumor growth. The* Salmonella* type III secretion system (TTSS) has been extensively used to deliver tumor antigens [64–66].* Salmonella* TTSS is a needle-like structure which contains a sensory probe to detect the presence of eukaryotic cells and directly inject proteins (effectors) into host cells, thus making it an effective antigen translocation candidate [54]. Other secretion system and surface structures have also been used to design tumor-antigen presenting strains. For instance, the antigen C-Raf (a serine-threonine kinase) was expressed by an attenuated* S. typhimurium aroA* strain using the* E. coli* hemolysin secretion system [67]. The C-Raf expressing strain significantly reduced tumor growth in two transgenic mouse models of Raf oncogene-induced lung adenomas [67]. Fensterle et al. [68] have used the* S. typhimurium aroA* strain type I secretion system in combination with cholera toxin subunit B to deliver PSA (prostate-specific antigen)* in vivo*. The strain was found to induce cytotoxic CD8+ T-cell responses resulting in efficient prevention of tumor growth in mice [68]. The* Salmonella* fimbrial display system has been used to express NY-ESO-1 p157–165 or p157–167 (T-cell epitopes) to induce NY-ESO-1 (a human cancer antigen) p157–165-specific CD8(+) T cells* in vivo* [69].

### 4.4. Transferring Eukaryotic Genes and Expression of Oncogene Silencing RNA

*Salmonella* has been shown to transfer eukaryotic expression plasmids to mammalian host cells* in vitro* and* in vivo* [70]. Some cytotoxic agents, cytokines, and tumor antigens have been designed to be expressed in tumor cells by* Salmonella* transfection [42]. However, this strategy is far from perfect due to its uncontrollability and low efficiency. Recently, oncogene silencing RNA has been introduced into tumor-targeting* Salmonella*. Zhang et al. [71] reported that STAT3-specific siRNAs expressed by attenuated* S. typhimurium* significantly inhibited tumor growth and metastasis and extended the life of C57BL/6 mice bearing a prostate tumor compared to bacterial treatment alone. Similarly, a short hairpin RNA (shRNA) targeting the tolerogenic molecule STAT3 encoded by* Salmonella* also increased apoptosis in tumors of treated mice, enhancing tumor-specific killing of tumor targets [72].

### 4.5. Engineered Tumor-Targeting* Salmonella* as Tumor-Detection Tools

The preferential tumor-targeting and accumulation phenotype coupled with genetic tools for strain reengineering made* Salmonella* a good tumor-detection tool. Fluorescent proteins such as GFP have been used to label and visualize* Salmonella* as well as other tumor-targeting bacterial strains* in vivo* [18, 73, 74]. These fluorescent proteins are good for whole-mouse imaging but may not be suitable for use in the human body due to thick tissues. Magnetic resonance and positron emission have recently been used to detect bacteria that accumulated in tumors. For example, fluorine-19 magnetic resonance spectroscopy has been tested to monitor the conversion of 5-fluorocytosine (5-FC) to 5-fluorouracil (5-FU) using an attenuated* S. typhimurium* strain recombinant to provide cytosine deaminase (TAPET-CD)* in vivo* [51]. VNP20009 has been engineered to express the herpes simplex thymidine kinase (HSV1-tk) reporter gene that can selectively phosphorylate radiolabeled 2′-fluoro-1-beta-D-arabinofuranosyl-5-iodo-uracil (FIAU) [75]. PET images could identify multiple tumor sites using this reporter strain [75]. Recently, Panteli et al. [76] engineered attenuated* Salmonella* to express the fluorescent protein ZsGreen. Then, a specific antibody was employed to detect bacterially produced ZsGreen. This system significantly increased sensitivity and could detect tumors 2600 times smaller than the current limit of tomographic techniques. These results indicate that the noninvasive* Salmonella* vectors have the potential to be used in clinical applications to either diagnose or cure tumors.

## 5. Unsolved Problems and Perspectives

Although* Salmonella*-based tumor therapy is a promising candidate for treating cancer, there are still many problems that need to be solved. The key issues are* Salmonella* strain toxicity and improving* Salmonella*-mediated destruction of tumors. Additionally, tumor-targeting specificity can still be improved. Selection, massive strain screening, and genetic manipulation can solve these issues.* Salmonella* can be developed to deliver antitumor molecules or drugs to enhance their tumor toxicity. Combination therapies that include tumor-seeking* Salmonella* and traditional tumor therapy may enhance the curative effects in a synergistic fashion. Inducible promoters may improve our “*Salmonella* bomb” to destroy tumors in an accurate time point and a proper place [77–79].

Cancer is a highly complicated disease. Genetic and phenotypic profiles vary among different tumor types that often result in different responses to therapy. Whether a universal therapeutic* Salmonella* strain can be developed that can treat most if not all tumor presentations is unknown. However,* S. typhimurium* has been shown to effectively treat prostate, breast, pancreatic, and spinal-cord cancers, as well as some tumor metastases, with a moderate success rate [19]. The wide spectrum of tumor types targetable by* Salmonella* gives direction for improving bacterial cancer therapy by combining the* Salmonella* tumor-targeting vector with highly effective chemotherapies that cannot target and accumulate at tumors in high local concentrations. Elucidation and improvement of* Salmonella* tumor-targeting mechanism(s) using the VNP20009, A1-R, and CRC2631 model organisms will improve and speed optimization of these therapies. Bacterial-based tumor therapy may not replace all tumor treatment methods but will provide us with another valuable tool to combat cancer.
